# Identification of anthranilate and benzoate metabolic operons of *Pseudomonas fluorescens *and functional characterization of their promoter regions

**DOI:** 10.1186/1475-2859-5-1

**Published:** 2006-01-05

**Authors:** Diane M Retallack, Tracey C Thomas, Ying Shao, Keith L Haney, Sol M Resnick, Vincent D Lee, Charles H Squires

**Affiliations:** 1 The Dow Chemical Company, Biotechnology Research and Development, 5501 Oberlin Dr. San Diego, CA 92121, USA

## Abstract

**Background:**

In an effort to identify alternate recombinant gene expression systems in *Pseudomonas fluorescens*, we identified genes encoding two native metabolic pathways that were inducible with inexpensive compounds: the anthranilate operon (*antABC*) and the benzoate operon (*benABCD*).

**Results:**

The *antABC *and *benABCD *operons were identified by homology to the *Acinetobacter sp*. anthranilate operon and *Pseudomonas putida *benzoate operon, and were confirmed to be regulated by anthranilate or benzoate, respectively. Fusions of the putative promoter regions to the *E. coli lacZ *gene were constructed to confirm inducible gene expression. Each operon was found to be controlled by an AraC family transcriptional activator, located immediately upstream of the first structural gene in each respective operon (*antR *or *benR*).

**Conclusion:**

We have found the anthranilate and benzoate promoters to be useful for tightly controlling recombinant gene expression at both small (< 1 L) and large (20 L) fermentation scales.

## Background

Ideally, to facilitate control of gene expression for production of proteins in an organism like *P. fluorescens*, which has been developed as a robust recombinant protein expression system[1,2], it is desirable to have a collection of expression cassettes. These cassettes would contain a variety of promoters that are tightly regulated, of differing strengths, induced under different growth conditions, and/or by different chemicals. These expression cassettes can then be linked to various genes of interest to achieve total control of those genes under typical fermentation conditions. High levels of gene expression are often obtained in the *P. fluorescens *system using the *E. coli lac*UV5 and *tac *promoters [1]. Several bacterial promoters have been previously shown to be effective at driving transgene expression in pseudomonads including the bacteriophage λ P_R _and P_L _promoters [3,4], which are regulated by a temperature sensitive repressor protein, and the *Pseudomonas *Pm, Pu, and Psal promoters [4,5], which are regulated by alkyl- or halotoluenes (Pm and Pu) or salycilates (Psal) and the T7 early promoter [6], regulated by isopropyl-thiogalactopyranoside (IPTG).

Pseudomonads are capable of metabolizing a wide variety of aromatic hydrocarbons, including benzoate and anthranilate [7-11], which are inexpensive and non-toxic. Benzoate and anthranilate are converted to catechol by benzoate 1,2-dioxygenase together with 2-hydro-1,2dihydroxybenzoate dehydrogenase, and anthranilate 1,2-dioxygenase respectively. These enzymes are encoded by the *benABCD *and *antABC *operons, which have been identified in several organisms [9-16], and are often regulated by transcriptional activators belonging to the AraC/XylS family of transcriptional regulators. Transcriptional activators BenR and BenM have been described, which activate transcription of the *benABCD *operon [7,17,18]. Recently transcriptional activators of two different anthranilate operons have been described [10,13].

We describe in this report the identification of the *P. fluorescens *strain MB214 *benABCD *and *antABC *operons, along with the genes coding for the transcriptional regulatory proteins BenR and AntR, respectively. The promoter regions of the *benABCD *and *antABC *operons have been defined, and regulation by BenR and AntR examined. A range of potential inducing compounds, in addition to benzoate and anthranilate, were tested including *o*-toluate, *m*-toluate, *p*-toluate and chlorinated anthranilates. Promoter elements are defined which are capable of driving heterologous gene expression at both small (1 L shake flask) and large (20 L fermentor) scales.

## Results

### *P. fluorescens *MB214 can utilize anthranilate and benzoate as sole carbon source

To characterize the functional catabolic and regulatory potential of *Pseudomonas fluorescens *strain MB214 (Table 1), the strain was tested for the ability to utilize a variety of aromatic and aliphatic hydrocarbons or aromatic acids as sole sources of carbon for growth. Carbon substrates were supplied at 5–10 mM as sole source of carbon and energy in liquid minimal medium (M9 containing 1 mM MgSO_4 _and 5 mg/ml trace metals solution), unless stated otherwise. Growth in liquid media was measured spectrophotometrically at 600 nm. In most cases, growth was also examined on solid media of identical composition but containing 1.5% (w/v) agar. Naphthalene and toluene were supplied in the vapor phase to agar plates containing carbon-free M9 medium. Growth of strain MB214 in media containing test substrates was compared to that observed in control media lacking a carbon test substrate. Results of the phenotypic screens showed that benzoate, anthranilate, 1-decanol, and 1-dodecanol were able to support growth of *P. fluorescens*. However, salicylate, phenylacetate, phthalate, phenol, naphthalene, *n*-octane, *n*-decane, *n*-dodecane, *n*-hexadecane and toluene were not able to support grow of *P. fluorescens*.

**Table 1:** Bacterial strains used in this study

**Genus, Species**	**Strain Name**	**Relevant Genotype**
*Escherichia coli*	JM109	*lacZ*^-^
*Pseudomonas fluorescens*	MB101	*lacZ*^-^
*Pseudomonas fluorescens*	MB214	*lacZ*^+^
*Pseudomonas fluorescens*	DC164	*lacZ*^- ^Δ*pyrF*
*Pseudomonas fluorescens*	DC253	*lacZ*^- ^Δ*benAB*
*Pseudomonas fluorescens*	DC284	*lacZ*^- ^*benR*::pCR2.1

Subsequent experiments conducted in M9 media with glucose, glycerol, citrate, or succinate as primary growth substrates indicated that benzoate and anthranilate catabolism in strain MB214 was inducible by addition of the individual aromatic acid substrates. The functional evidence for catabolic pathways inducible by benzoate and anthranilate, or their metabolic intermediates, led to a systematic isolation and evaluation of the corresponding regulatory switches for the development of gene expression systems.

### Characterization of the anthranilate and benzoate metabolic operons

**Figure 1:**
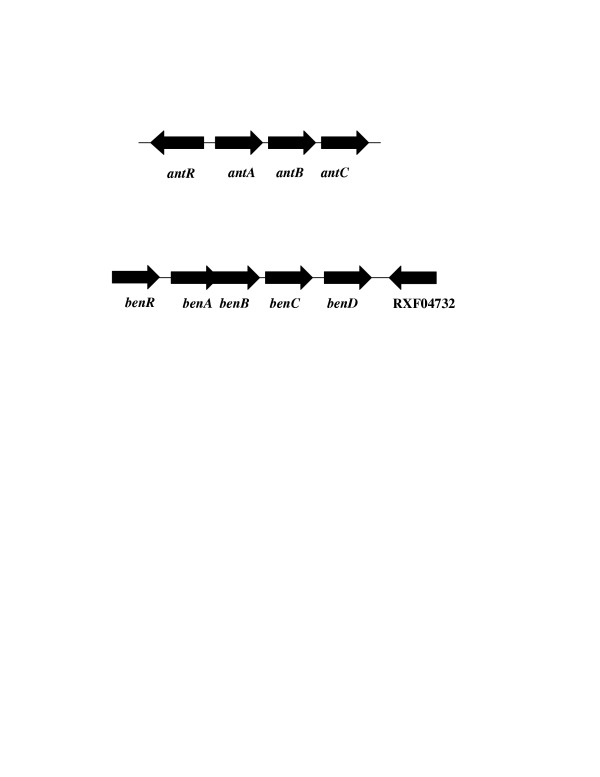
**Anthranilate and benzoate operons**. Above are depicted schematic representations (not drawn to scale) of the *P. fluorescens *MB214 anthranilate (*antR antABC *Genbank accession number DQ172833) and benzoate (*benR benABCD *Genbank accession number DQ172832) metabolic operons. Open reading frames for each gene are indicated by arrows, with the direction of the arrow corresponding to the direction of the open reading frame. Gene names are indicated below each arrow.

Sequence analysis and annotation of the *P. fluorescens *MB214 genome in comparison to public databases revealed putative anthranilate and benzoate operons similarly structured to the genes encoding the dioxygenase and reductase subunits clustered in one operon downstream of an AraC/XylS type transcriptional activator, likely driven by its own promoter (Figure 1). A second putative transcriptional activator, RXF04732, was identified downstream of the putative *benABCD *operon as well. As described later in this report, it is the upstream transcriptional activator that acts on the Pben promoter. The genes encoding the putative transcriptional activators, *antR *and *benR*, are orientated differently with respect to each dioxygenase operon (see Figure 1). Similar to other AraC/XylS type regulators and the recently described *Burkholderia cepacia *DBO1 *andR *gene [13], *antR *is oriented divergently from the putative *antABC *operon. However, *benR *is oriented in the same direction as *benABCD*, similar to the *benR *gene of *Pseudomonas putida *[7].

The proteins coded by the anthranilate and benzoate operons were compared to the UniProt protein database (01/05) using the BLASTP program [19]. A comparison of the AntA, AntB and AntC proteins to homologues of *P. aeruginosa*, *P. putida *and *P. resinovorans *is shown in Table 2. The *P. fluorescens *MB214 AntA, AntB, AntC and AntR proteins, were most similar to those *P. resinovorans*. Analysis of *P. fluorescens *BenA, BenB, BenC, BenD and BenR protein to homologues of *P. aeruginosa *and *P. putida *is shown in Table 3. These proteins were found to be homologous to those coded by the *P. aeruginosa *toluate 1,2-dioxygenase and *P. putida *benzoate catabolic operons. The similarity of these proteins to those coded for by previously identified catabolic operons, together with the functional characterization of anthranilate and benzoate metabolism, indicate that these operons likely code for the anthranilate and benzoate operons of *P. fluorescens*.

**Table 2:** Anthranilate operon homologues. Percent amino acid identity/similarity shown below. AntA: *P. aeruginosa *(UNI_TREMBL:Q9I0X0); *P. putida *(UNI_TREMBL:Q93SR3),, and *P. resinovorans *(GenPept: NP_758565). AntB: *P. aeruginosa *(UNI_TREMBL:Q9I0SR8); *P. putida *(UNI_TREMBL:Q90W9); *P. resinovorans *(GenPept: NP_758547). AntC: *P. aeruginosa *(UNI_TREMBL:Q90W8); *P. putida *(UNI_TREMBL:Q93SR4), *P. resinovorans *(GenPept: NP_758546). Anthranilate dioxygenase transcriptional activator AntR: *P. resinovorans *(GenPept: NP_758551).

	*P. aeruginosa *homologue (%identity/similarity)	*P. putida *homologue (identity/similarity)	*P. resinovorans *homologue (%identity/similarity)
*P. fluorescens *AntA	80/90	45/66	91/95
*P. fluorescens *AntB	71/81	71/82	74/85
*P. fluorescens *AntC	63/77	63/77	74/84
*P. fluorescens *AntR			74/86

**Table 3:** Benzoate operon homologues. Percent amino acid identity/similarity shown below. BenR: XylS of *P. aeruginosa *(UNI_TREMBL:Q9I0W3) and *P. putida *(UNI_SPROT:XYS3_PSEPU). BenA: *P. aeruginosa *toluate 1,2 dioxygenase alpha subunit (UNI_TREMBL:Q9I0W4); *P. putida *BenA (UNI_TREMBL:Q88I40). BenB: *P. aeruginosa *toluate 1, 2-dioxygenase small subunit (UNI_TREMBL:Q9I0W5); *P. putida *BenB (UNI_TREMBL:Q88I39). BenC: *P. aeruginosa *toluate 1,2 dioxygenase subunit (UNI_TREMBL:Q9WWV0); *P. putida *BenC (UNI_TREMBL:Q88I38). BenD: *P. aeruginosa *cis 1,2-dihydroxycyclohexa-3,4-diene carboxylate dehydrogenase (UNI_TREMBL:Q9I0W7); *P. putida *BenD (UNI_TREMBL:Q88I37).

	*P. aeruginosa *homologue (% identity/similarity)	*P. putida *homologue (% identity/similarity)
*P. fluorescens *BenA	64/79	64/79
*P. fluorescens *BenB	66/78	70/79
*P. fluorescens *BenC	53/68	53/68
*P. fluorescens *BenD	77/84	75/82
*P. fluorescens *BenR	58/73	54/70

### Isolation of the Pant and Pben promoters

**Figure 2:**
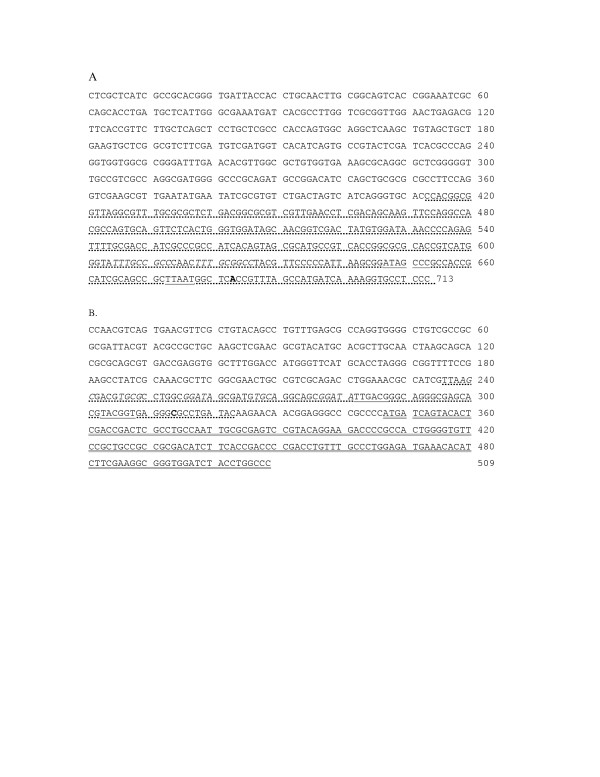
**Anthranilate (A) and benzoate (B) promoter regions**. The largest promoter region tested for each is shown. Pant311 = bases 413–713 (A, dotted underline), Pben278 = bases 236–509 (B), Pben87 = bases 236–323 (B, dotted underline). Underlined with solid lines are the putative -35 and -10 sites. A 9 bp direct repeat is show in italics (A). Putative XylS- type activator binding sites are shown in italics (B). The transcriptional start for each promoter is shown in bold. Double underlined in B is 5' end of the *benA *coding region.

**Figure 3:**
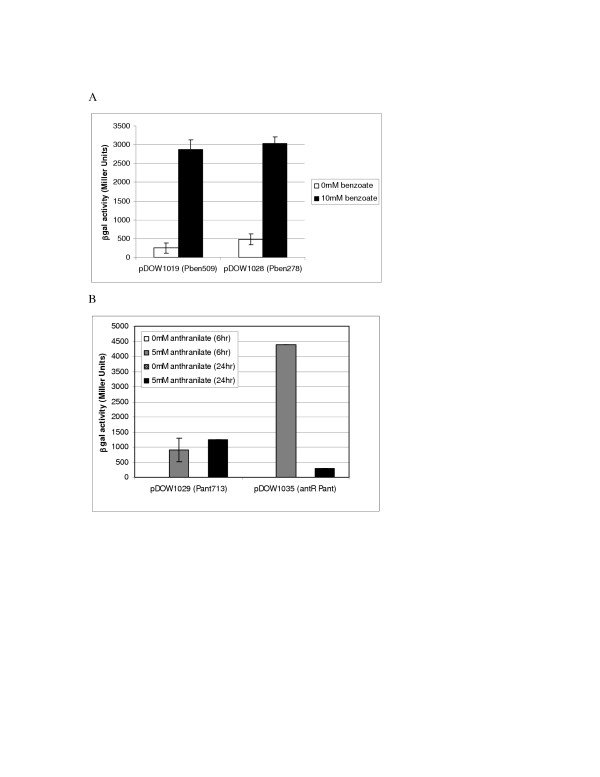
**Anthranilate and benzoate promoter activity**. Cultures were grown in M9 medium supplemented with 1% glucose. Induction of Pben promoter constructs for 24 hours is shown in panel A. Induction of Pant promoter constructs for 6 (grey bars) or 24 (black bars) hours is shown in panel B. β-galactosidase activity for each is depicted on the Y-axes. Promoter construct tested is indicated on the X-axis. White or hatched bars indicate uninduced samples and grey or black bars indicate induced samples. Standard deviation of triplicate wells is depicted by error bars. A representative experiment is shown.

Initially, approximately 500–700 bp regions upstream of the *antA *and *benA *genes (Figure 2) were amplified and cloned upstream of the *lacZ *reporter gene in pDOW1017. As shown in Figure 3A and 3B the resulting plasmids, pDOW1019 and pDOW1029 are found to contain benzoate and anthranilate inducible promoters, respectively. Uninduced cultures showed low level β-galactosidase activity in the presence of the Pben::*lac*Z fusion. However, no β-galactosidase activity was detected in uninduced cultures carrying the Pant::*lacZ *fusion. The Pben and Pant promoter containing fragments were next truncated to include only the region between the putative translation start of the first gene of the structural operon (*benA *and *antA *respectively) and the upstream putative transcriptional activator open reading frame (ORF). The truncated benzoate promoter fragment, Pben278, was amplified and cloned upstream of the *lacZ *reporter gene in pDOW1017. The *P. fluorescens *strain MB101 (Table 1) was transformed with the resulting construct, pDOW1028, and induced with benzoate to assess activity. Figure 3A shows that Pben278 exhibits benzoate inducible activity similar to Pben509, indicating that the 275 bp truncated promoter contains all elements necessary for benzoate-activated transcription. Moreover, the Pben fragment was further truncated at the 3' end to just 87 bp based on primer extension mapping of the transcriptional start site as described below. Similar to Pben278, Pben87 was also found to be as active as the original Pben509 fragment (data not shown). Refined annotation of the 5' region of the *benA *gene showed the translation start to be 25 base pairs downstream of the 3' end of Pben87 (see Figure 2). The truncated anthranilate promoter fragment, Pant311, was amplified and cloned upstream of the *lacZ *reporter gene of pDOW1017. Little activity was detected from the truncated promoter when fused to the *lacZ *reporter gene (data not shown).

### Overexpression of transcriptional activator increases Pant activity

In an attempt to improve the activity of the anthranilate inducible promoter, the promoter fragment was amplified to include the putative transcriptional activator ORF directly upstream of *antA*. This PCR fragment was cloned upstream of the *lacZ *reporter gene of pDOW1017. MB101 was transformed with the resulting plasmid, pDOW1035, and activity upon induction with anthranilate was assessed. The addition of the transcriptional activator in multicopy demonstrated faster and greater induction compared to Pant713 (Figure 3B), which does not contain the entire transcriptional activator ORF. The activity of the culture harbouring pDOW1035 peaks early, then declines between 6 and 24 hours. This is likely due to the rapid decline in anthranilate concentration, compared to the culture carrying pDOW1029 (data not shown). Similar experiments were performed with a fragment containing *benR *and the Pben509 fragment, however no difference in promoter activity were observed (data not shown).

### Inactivation of the *benABCD *operon transcriptional activator

**Figure 4:**
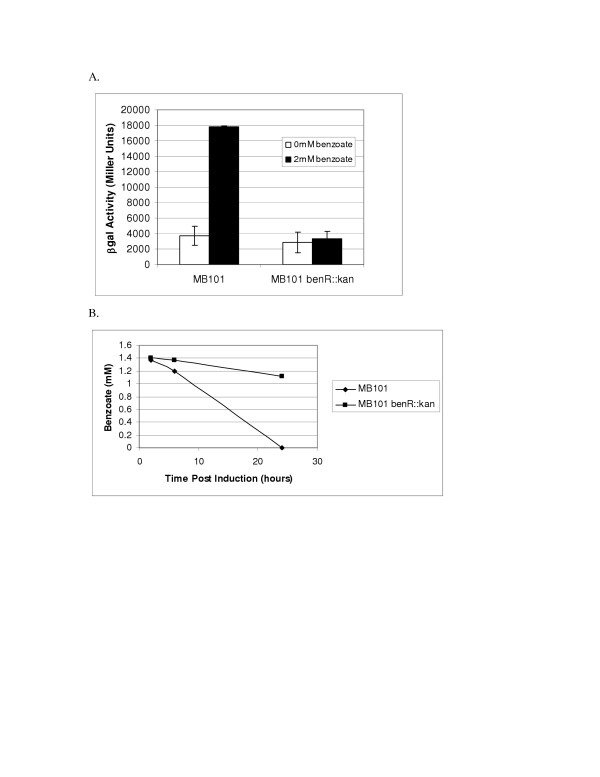
**Effect of *benR *inactivation on Pben278 activity**. Above is shown β-galactosidase activity (A) of either wild type (MB101) or *benR *inactivated (MB101 *benR*::kan) carrying pDOW1019. Cultures were grown in M9 medium supplemented with 1% glucose and induced with 2 mM benzoate for 24 hours. Standard deviation of triplicate wells is depicted by error bars. A representative experiment is shown. Benzoate metabolism over the course of the induction is shown in B, with benzoate concentration shown on the Y-axis and time post induction shown on the X-axis.

There are two putative transcriptional regulator coding sequences that flank the *benABCD*. To determine if the ORF *benR*, upstream of *benABCD*, acts as the benzoate transcriptional activator, we insertionally inactivated *benR *as described in Materials and Methods. Following inactivation of the putative benzoate transcriptional activator, the resultant strain DC284 was transformed with the Pben::*lacZ *fusion, pDOW1019, and tested for benzoate activated *lacZ *expression. As shown in Figure 4A, inactivation of *benR *results in an inability to activate Pben::*lacZ *expression. Moreover, benzoate is not metabolized as in the wild-type MB101 strain (Figure 4B) indicating that the chromosomal benzoate dioxygenase operon (*benABCD*) also fails to be induced in the transcriptional activator knockout strain.

### Alternative inducers of Pant and Pben promoters

**Figure 5:**
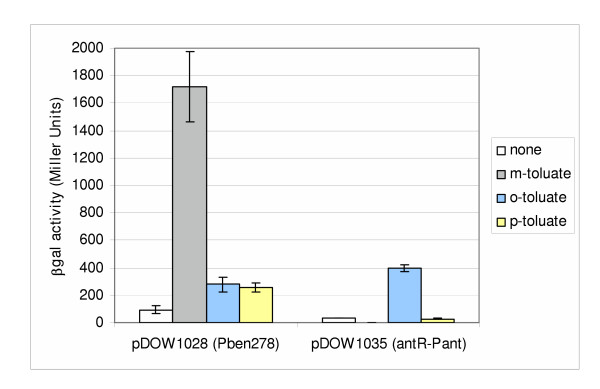
**Induction of *antR*-Pant and Pben278 by toluate**. Shown on the X-axis is β-galactosidase activity of Pben278 and *antR*-Pant constructs (pDOW1028 and pDOW1035 respectively) 8 hours post-induction. Cultures were grown in M9 medium supplemented with 1% glucose. The inducer (5 mM) used in each case is as described in the legend to the right of the bar graph. Standard deviation of triplicate wells is depicted by error bars. A representative experiment is shown.

*Ortho*-, *meta*- and *para*-toluate were tested as inducers of Pant and Pben. Similar to other systems [11,16], *m*-toluate was found to be an inducer of the *P. fluorescens *benzoate operon promoter. Addition of 5 mM *m*-toluate induced Pben278 (pDOW1028) to significant levels, ~13 fold over background (Figure 5). However, addition of 5 mM *o*-toluate and *p*-toluate induced Pben278 only slightly. The *antR*-Pant construct (pDOW1035) was induced by 5 mM *o*-toluate approximately 19- fold over background, although to levels still lower than *m*-toluate induction of Pben (Figure 5). *P. fluorescens *growth on solid medium consisting of 1XM9 salts supplemented with either *m*-, *o*-, or *p*-toluate (5 mM) as a sole carbon source was tested in comparison to growth on similar solid medium supplemented with 0.5% glucose as a carbon source. While *P. fluorescens *grew on medium supplemented with glucose, growth on *m*-, *o*-, or *p*-toluate as carbon source was not observed even after 14 days of incubation (data not shown). These results indicated that although *m*-, *o*- and *p*-toluate could not be utilized as carbon sources, certain forms of toluate can act as significant inducers of the Pben278 and *antR*-Pant promoter fusions.

**Figure 6:**
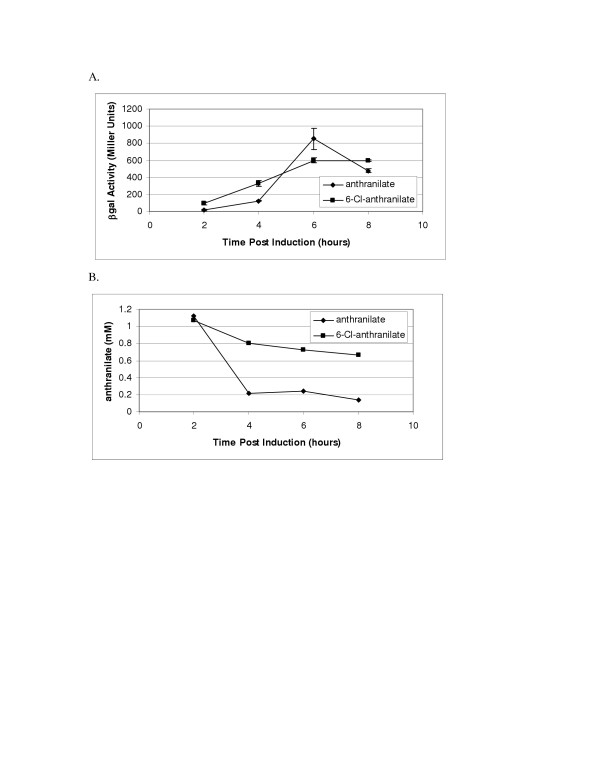
**Induction of antR-Pant by 6-chloro-anthranilate**. Above is shown a time course of Pant induction (MB101 carrying pDOW1035) performed in M9 media supplemented with 1% glucose. Cultures, induced with either 2 mM anthranilate (diamonds) or 2 mM 6-chloro-anthranilate (squares), were monitored for β-galactosidase activity (A) and inducer concentration (B) as shown on the Y-axes. Time post induction in hours is shown on the X-axes. Standard deviation of triplicate wells is depicted by error bars. A representative experiment is shown.

Analysis of anthranilate catabolism in strain MB214 revealed that several chloro-anthranilates acted as inducers of the anthranilate catabolic pathway (data not shown). The pDOW1035 construct containing *antR*-Pant, which showed higher expression levels than pDOW1029 (Pant713), was used to compare Pant induction by 2 mM 6-chloro-anthranilate to that by 2 mM anthranilate. As shown in Figure 6A, the level induction of the *antR*-Pant::*lacZ *fusion by 6-chloroanthranilate was similar to that measured with anthranilate. Induction with 6-chloroanthranilate showed a steady increase in promoter activity over time, which appeared to level between 6 and 8 hours. Induction with anthranilate showed a rapid increase in promoter activity between 4 and 6 hours, which then declined from 6 to 8 hours. The decline in activity was likely to due to metabolism of the inducer. Figure 6B shows concentration of the inducing compounds throughout the time course of induction. While the concentration of 6-chloroanthranilate decreased slightly during the time course, the concentration of anthranilate decreased significantly. These results indicate not only that 6-chloroanthranilate can act as an inducer of Pant, but that it may act as a gratuitous inducer, as it does not appear to be metabolized significantly by *P. fluorescens*.

### Mapping transcriptional start sites of Pben and Pant promoters

**Figure 7:**
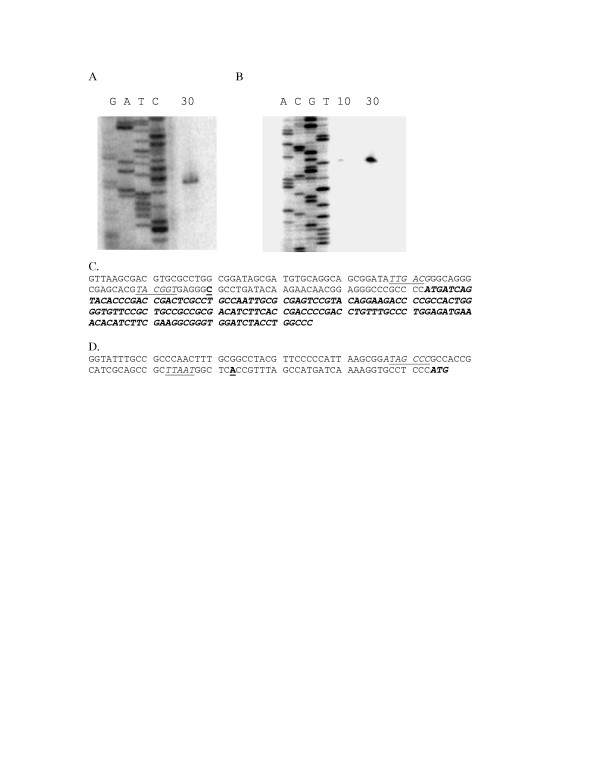
**Mapping transcriptional start sites of Pben and Pant promoters**. Shown above (A) are primer extension products of 30 μg RNA isolate from MB101 carrying pDOW1028 (Pben278) induced with benzoate run next to the sequence ladder generated with the same primer (GATC). Shown in (B) is the primer extension product of 10 or 30 μg RNA isolated from MB101 carrying pDOW1029 (Pant713) induced with anthranilate is run to the right of a sequence ladder (ACGT) generated with the same primer. The DNA sequence of the Pben278 promoter is shown in (C). with the bold, underlined C indicating the transcriptional start site. The putative -35 and -10 regions are shown in italics and underlined and the 5' portion of the *benA *coding sequence is shown in bold italics. The 3' portion of the Pant promoter fragment is show in (D), with the transcriptional start site indicated in bold and underlined and the translational start in bold italics. The putative -35 and -10 regions are shown in italics and underlined.

Total RNA was isolated from benzoate induced cultures of MB101 carrying either pDOW1019 (Pben509) or pDOW1028 (Pben278). Total RNA from each was subjected to primer extension analysis using the lacZPE primer. Both constructs show the transcriptional start at a cystine nucleotide 196 bp upstream of the 3' end of the Pben278 clone (Figure 7A). This is consistent with the location of the putative -35 and -10 promoter sequences shown in italics and underlined in Figure 7C.

Total RNA was isolated from an anthranilate induced culture of MB101 carrying pDOW1029 (Pant713). Primer extension analysis was performed using the lacZPE2 primer. An extension product was observed corresponding to the adenosine nucleotide 31 bases upstream of the 3' end of the Pant713 clone (Figure 7B). This start site is consistent with the spacing of the putative -10 and -35 promoter sequences shown in italics and underlined in Figure 7D.

### Analysis of Pben and Pant activity at the 20 L fermentation scale

**Figure 8:**
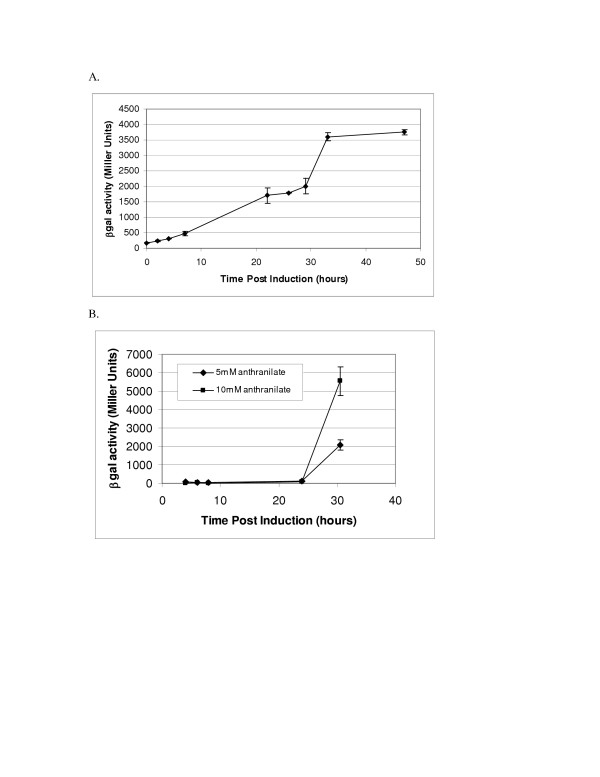
**Promoter activity at the 20 L scale**. Induction of Pben278:*lacZ *with ~6 mM benzoate (A) and induction of antR-Pant:*lacZ *with 5 or 10 mM anthranilate (B) are shown above. β-galactosidase activity is shown on the Y-axes, and time post induction in hours is shown on the X-axes. Standard deviation of triplicate wells is depicted by error bars. A representative experiment is shown.

*P. fluorescens *MB101 carrying the plasmid pDOW1028 (Pben278 promoter) was grown in a standard 20 L fermentation, as described in Materials and Methods, and induced with 6 mM sodium benzoate. Promoter activity was monitored as a function of *lacZ *expression over the time course of the fermentation. However, under standard conditions using glucose as a carbon source, no promoter activity was observed. This was not consistent with results using glucose as a carbon source shown at the shake flask scale (Figure 3). Additional shake flask experiments revealed that the Pben278 promoter was active when either citrate or glycerol was used as a carbon source as well (data not shown). When glycerol was used in place of glucose as a carbon source for 20 L fermentation, Pben278 activity was observed following induction with benzoate, and increased over the course of the fermentation (Figure 8A). The level of induction was comparable to that observed at the shake flask scale.

The *antR*-Pant construct pDOW1035 was also tested at the 20 L scale. MB101 carrying pDOW1035 was grown under standard fermentation condition, with glucose as a carbon source. Following induction with either 5 mM or 10 mM anthranilate, promoter activity was observed (Figure 8B). The culture induced with 10 mM anthranilate showed a higher level of activity than that induced with 5 mM anthranilate, as expected. Activity was not detected until very late in the fermentation unlike Pben278, which showed an increase in activity between 6 and 24 hours (Figure 8A).

### Benzoate induces Pben509

**Figure 9:**
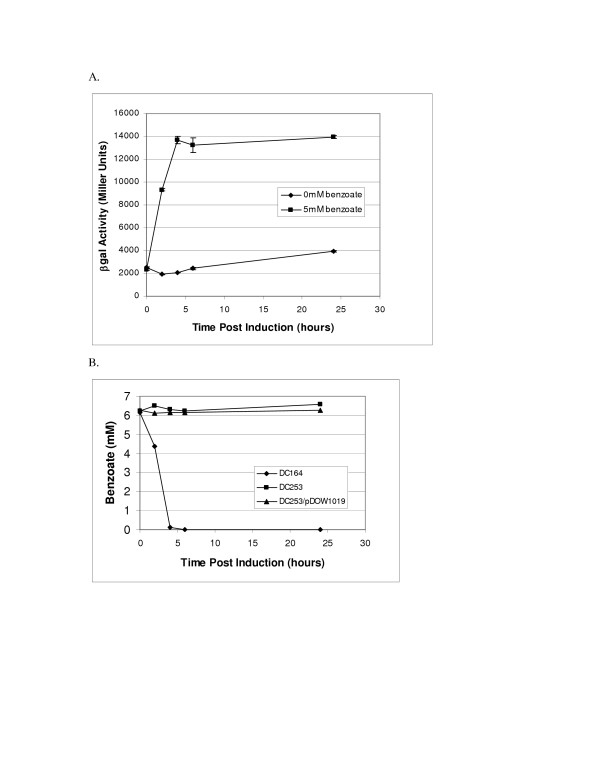
**Analysis of Pben509 activity in a Δ*benAB *strain**. Shown is β-galactosidase activity of DC253 carrying pDOW1019 (Pben509::*lacZ*) uninduced (0 mM benzoate) or induced with 5 mM sodium benzoate (A). Standard deviation is depicted by error bars. Cultures were grown in LB medium supplemented with uracil and proline. Benzoate metabolism in the parental strain (DC164, Δ*pyrF*) compared to the Δ*benAB *strain (DC253, Δ*pyrF*Δ*benAB*) is shown in B.

To determine whether benzoate is in fact the inducer of the Pben509 promoter and not a downstream metabolite, we sought to block the metabolism of benzoate at the first step by deleting the *benA *and *benB *genes, which code for the large and small subunits of benzoate 1, 2-dioxygenase respectively. A deletion of *benAB *was constructed in the Δ*proC*Δ*pyrF *strain DC164 as described in Materials and Methods. The resultant strain, DC253, was tested for the ability to metabolize benzoate and for the ability to support benzoate induced β-galactosidase activity from the Pben509::*lacZ *construct. To test Pben activity, DC253 was transformed with pDOW1019 and the resultant strain was induced with 5 mM benzoate. As shown in Figure 9A, Pben509::*lacZ *remains active in the *benAB *knockout strain, *i.e*. β-galactosidase activity is detected. The concentration of benzoate remaining in the cell-free broth as measured by HPLC showed that the *benAB *deletion mutants were unable to metabolize benzoate, while the parent strain DC164 did metabolize benzoate efficiently (Figure 9B). These results indicate that benzoate does indeed act as an inducer Pben509, and not necessarily a downstream metabolite. This conclusion is consistent with the observation that Pben509 was not induced by cis-cis-muconate (data not shown).

## Discussion

The work presented here describes the anthranilate and benzoate catabolic operons of *P. fluorescens *strain MB101. The anthranilate dioxygenase large and small subunits (AntA and AntB) and reductase (AntC) are most closely related to the anthranilate dioxygenase and reductase recently described in *P. resinovorans*. Although the transcriptional activator, AntR, is also closely related to the transcriptional activator of *P. resinovorans*, the chromosomal arrangement of *P. fluorescens antR *gene in relation to the *antABC *is different than that of *P. resinovorans *[10]. The *P. fluorescens antR *gene is divergently transcribed from the *antABC *operon, similar to the arrangement of the anthranilate dioxygenase operon of *B. cepacia *[13]. The *P. fluorescens *AntR represents further evidence of an AraC/XylS type transcriptional activator regulating anthranilate dioxygenase expression. Transcriptional activators for anthranilate operons have only recently been described [10,13]. Studies of *antABC *promoter activation presented here indicate that AntR may be the limiting factor in expression from the Pant promoter. Addition of *antR *in multicopy results in significant improvement of Pant activity (Figure 3) indicating that the presence of presumably higher levels of inducer alone was not sufficient to promote high levels of gene expression from the Pant promoter. It is possible that either AntR is produced at low levels, or that the protein binds weakly to the promoter region. In either case, increasing the overall concentration of the protein would result in improved promoter activity. The rapid metabolism of anthranilate in the pDOW1035 harbouring strain supports the observation that overexpression of *antR *results in improved Pant activity, since increased activity of the chromosomally located Pant promoter would result in increased expression of the anthranilate dioxygenase, and subsequently, increased anthranilate metabolism. Although AntR shows high homology to other AraC/XylS transcriptional activators, we were unable to detect a consensus XylS type DNA binding site (TGCA-N6-GGNTA) [20] upstream of the Pant core promoter. Further investigation of AntR expression and elucidation of its binding site in the Pant promoter may help to improve promoter activity. The Pben promoter fragment, however, does appear to have a XylS type DNA binding site as shown in italics in Figure 2B. The sequences TGCG-N6-GGATA and TGCA-N6-GGATA, separated by 5 bp, are located immediately upstream of the putative -35 signal. Further investigation is required to determine whether these sequences are important for Pben activation. Unlike the *antR*-Pant system, increasing the copy number of *benR *did not improve Pben activity (data not shown).

Anthranilate and benzoate have been shown to be inducers of the Pant and Pben promoters, respectively, as expected. Not unexpectedly, several other compounds were found to induce the promoters. *m*-toluate, which has been shown to induce other benzoate operons, including those of *Rhodococcus *sp. strain 19070 [16] and *P. putida *mt-2 [11], was found to induce the Pben promoter 13-fold over background. *o*-toluate, which has not been previously reported to induce either benzoate or anthranilate operon promoters, was found to induce the Pant promoter 19-fold. However, *o*-toluate induction of the Pben promoter, along with *p*-toluate induction, was only 2.5 to 3-fold over background. The anthranilate promoter was also inducible with 6-chloro-anthranilate. The fact that 6-chloro-anthranilate was not metabolized significantly by *P. fluorescens *strain MB101 indicates that 6-chloro-anthranilate acts as a gratuitous inducer, and that anthranilate itself, and not necessarily a metabolite of anthranilate, may act as an inducer. Benzoate was shown to be the inducer of the Pben promoter by deleting the genes coding for the benzoate dioxygenase large and small subunits. As shown in Figure 9, the Pben promoter remains active in the absence of benzoate metabolism.

For Pben and Pant to be used as part of an expression system, it is important to identify inducing compounds that are not metabolized by the host organism. We have shown that the Pben and *antR*-Pant promoters can drive heterologous gene expression at the 20 L fermentation scale. Although the Pben promoter appears to be repressed under the glucose feed conditions used at the 20 L fermentations scale, the *antR*-Pant promoter construct was found to be active. However, a change in carbon source from glucose to glycerol alleviated the observed Pben repression at the 20 L scale. Additional fermentation optimization may result in improved promoter activity and heterologous gene expression. Further strain development, such as chromosomal deletion of the anthranilate and benzoate dioxygenases should also improve expression during the course of induction and simplify the induction process, in that the inducer will not need to be added more than once during the course of induction to maintain expression levels.

## Conclusion

It is important to identify and develop several recombinant expression systems to allow for flexibility and reliability in protein production. The availability of more than one inducible expression system will allow for differential expression of proteins during production. Differential expression of chaperones, foldases or disulfide bond isomerases from these alternate expression systems may aid in overall yield of active target protein. We have shown that the benzoate and anthranilate promoters examined in this work can be used to drive recombinant protein expression in *P. fluorescens *at both small (shake flask) and large (20 L) scales. Further development of these promoter systems will aid in expression of complex proteins in *P. fluorescens*.

## Methods

### Bacterial strains and growth conditions

Bacterial strains and plasmids used are listed in Tables 1 and 4. All plasmid construction and propagation was performed using JM109 (Promega, Madison, WI) as a host cell, functional assays were performed in *P. fluorescens *MB101 or derivatives of MB101 as described. The *P. fluorescens *strain MB214 is a derivative of MB101, which contains the *E. coli lacZYA *operon integrated into the genome. Tetracycline was used at a concentration of 15 μg/ml in both *E. coli *and *P. fluorescens *hosts; kanamycin was used at a concentration of 50 μg/ml in both *E. coli *and *P. fluorescens *hosts; ampicillin was used at a concentration of 100 μg/ml in *E. coli *hosts. Cells were grown on LB medium (Difco, Becton Dickenson, Sparks, MD), or when indicated minimal medium consisting of 1XM9 salts (Difco, Becton Dickenson, Sparks, MD), trace elements and either glucose, citrate or glycerol as a carbon source as indicated. *E. coli *strains were grown at 37°C, while *P. fluorescens *strains were grown at 30°C.

**Table 4:** Plasmids Used in This Study

**Plasmid**	**Relevant Feature**	**Source**
PCR2.1 TOPO	Cloning vector	Invitrogen
pNEB193	Cloning vector	New England Biolabs
pGEM-T Easy	Cloning vector	Promega
pDOW1017	Promoterless *lacZ*	This work
pDOW1019	Pben509::*lacZ*	This work
pDOW1020	*antR*-Pant	This work
pDOW1021	*benR*	This work
pDOW1022	Pben278	This work
pDOW1026	Pben509	This work
pDOW1028	Pben278::*lacZ*	This work
pDOW1029	Pant713::*lacZ*	This work
pDOW1033	Promoterless *phoA*	This work
pDOW1035	*antR *Pant::*lacZ*	This work
pDOW1041	Pben278::*phoA*	This work
pDOW1055	Pant311::*phoA*	This work
pDOW1081	Pben88::*phoA*	This work
pDOW1101	Pant311::*lacZ*	This work
pDOW1131	400 bp *benR *fragment	This work
pDOW1139	3' *benR *+ 5' *benC*	This work
pDOW1261-2	*tetAR pyrF*	This work

### 20 L fermentation

*P. fluorescens *fermentations were conducted in standard, aerated 20 L research fermentors with a mineral salts medium derived from Riesenberg, *et al*. [21]. Cultures were grown at 32°C and pH 6.5–7.0 was maintained through the addition of aqueous ammonia. Agitation and sparged airflow rates were gradually increased during the growth phase of the fermentation to control dissolved oxygen at a positive level (15% of saturation) but were fixed at maximum levels when these were reached. The cultures were operated as either a glucose or glycerol fed-batch or as a glycerol batch (glycerol concentration <200 g/L). The fermentation process was divided into an initial cell growth phase (typically 24–30 hours) and a gene expression (induction) phase in which inducer was added (anthranilate or sodium benzoate solution delivered as a bolus or fed) to initiate recombinant gene expression.

### General cloning methods

Restriction digestion, ligation and polymerase chain reaction were performed essentially as described in Sambrook and Russell. [22]. Restriction enzymes and modifying enzymes were purchased from New England Biolabs (Beverly, MA), and Taq polymerase was purchased from either Promega or Invitrogen (Carlsbad, CA). Genomic DNA was prepared using the Easy DNA isolation kit (Invitrogen) according to the manufacturer's protocol. Plasmid DNA was prepared using the Nucleospin or Nucleobond plasmid DNA kit (Clontech, Valencia, CA) according to the manufacturer's protocol. Primers used in this work are described in Table 5. *P. fluorescens *plasmids used in this study are derived from RSF1010 [23].

**Table 5:** Primers Used in This Study

**Primer Name**	**Sequence**
phoA5bam	CGGGATCCGGGCCTCCTTGCGGGT
phoA3Xho	CCGCTCGAGTTATTTCAGCCCCAGAGC
ant311	CCTTAATTAACAGGGTGCACCCACG
antPL+SD	CGGGATCCGGGAGGCACCTTTTGATCAT
antPL	CGGGATCCCTTTTGATCATGGCTAAC
antPU	GCTCTAGACTCGCTCATCGCCGCACG
AntAKO5	GGAATTCTTCGTGACGATGCG
AntAKO3	CGGGATCCGCTCGCGATGCTGC
lacZPE	GGATGTGCTGCAAGGC
lacZPE2	GTAACCATGGTCATCGC
ben40PU	CGGGATCCGGGCCAGGTAGATCCAC
ben40PL	CCTTAATTAACCAACGTCAGTGAACGTTC
benactko-for	CGCGACACATTGCTGCCCAG
benactko-rev	AGTATCAGCCATCGCACCTT
benL278	CCTTAATTAAGTTAAGCGACGTGCGC
3' antactiv	CCCAAGCTTCTATCGAGGCAAGCCAG
benact5'	AGCTTTGTTTAAACGCATGACGTTGTTGATTC
benact3'	CCCAAGCTTCCCGTCAATATCCGCTG
H3_5'benAKOclean	CCCAAGCTTGCCATGAGGCGGAAAACGCTGC
H3_3'benBKOclean	CCCAAGCTTCGGTGATCGCCACGCTGTCGC
benKOmega	CATACGTCATGGCCCTCCGTTGTTC
invbenKOmega	GAACAACGGAGGGCCATGACGTATG
5'benA_seq	CTGCTGGAAAACGCCTGCCTGGAG
seq_3'benB	GAGCACTTCAAGCATCGACAGGAAC
1261-8378F	CTTCAGATCCAGACTCACCAG
1261-103R	GACCATGATTACGCCAAGCGC
M13F	GTAAAACGACGGCCAGT
M13R	CACACAGGAAACAGCTATGAC

### Construction of benzoate promoter fusions

The Pben509 promoter fragment was amplified using the ben40PL and ben40PU primers with MB214 genomic DNA as template. The resulting fragment was digested with Pac I and BamH I and cloned into pDOW1017 to produce the Pben509::*lacZ *fusion pDOW1019. The Pben278 promoter fragment was amplified using primers benL278 and ben40PU, using pDOW1026 as template. PCR product was purified on a Microcon YM-100 column (Millipore, Billerica, MA). The PCR product was digested with Pac I and BamH I and cloned into the same sites of pNEB193 to produce pDOW1022. The BamH I-Pac I fragment of pDOW1022 containing the Pben278 fragment was then cloned into pDOW1017 to produce pDOW1028, a Pben278::*lacZ *fusion.

### Construction of anthranilate promoter fusions

The Pant713 Promoter was amplified using primers antPU and antPL and MB214 genomic DNA as the template. The resulting fragment was cloned into pDOW1017 to produce the Pant713::*lacZ *fusion pDOW1029. The Pant311 promoter was amplified using primers ant311 and antPL+SD using MB214 genomic DNA as the template. The PCR product was purified with a Microcon YM-100 column (Millipore) and the volume brought up to 50 μl with 5 mM Tris-Cl, pH8.0 (~100 ng/μl). The PCR product was cloned into the pGEM-T Easy vector (Promega) and sequenced using M13F and M13R primers. The pGEM clone was digested with BamH I and Pac I then cloned into the same sites of pDOW1033 creating a Pant311::*phoA *fusion, pDOW1055.

### Construction of promoter fusions with transcriptional activators

The putative benzoate operon transcriptional activator gene that lies upstream of *benA *was amplified using the primers benact5 and benact3, using MB214 genomic DNA as template. An ~1.2 kb fragment was isolated using the Prep-a-Gene kit (Biorad, Hercules, CA), digested with Pme I and Hind III and cloned into the same sites to pNEB193 (New England Biolabs) to produce pDOW1021. The Pme I – Hind III fragment of pDOW1021 was then cloned into the same sites of pDOW1019, upstream of the Pben509::*lacZ *fusion.

The anthranilate operon transcriptional activator that lies upstream of *antA *was amplified using the 3'antactiv primer and antPL+SD primer using MB214 genomic DNA as template. An ~1.3 kb fragment was purified using the Prep-a-Gene kit (Biorad), digested with Hind III and BamH I and cloned into the same sites to pNEB193 to produce pDOW1020. The BamH I -Hind III fragment of pDOW1020 was then cloned into the same site of pDOW1017 to produce pDOW1035.

### β-galactosidase assay

Strains of interest were grown overnight (30°C shaking at 250 rpm) in 1× M9 supplemented with 1% (w/v) glucose, 1 mM MgSO_4 _and trace elements. Strains were then subcultured and induced with indicated concentrations of anthranilate or benzoate. β-galactosidase activity of samples was analyzed in a 96-well format. For each sample well, buffer was prepared as follows: 152 μl Z buffer (0.06 M Na_2_HPO_4_-7H_2_O, 0.04 M NaH_2_PO4-H_2_O, 0.01 M KCl, 0.001 M MgSO_4_-7H_2_0) was mixed with 8 μl 1 M β-mercaptoethanol. Buffer was prepared in bulk quantities: to each 900 μl of this mix were added 1 drop 0.1%SDS and 2 drops CHCl_3_, and vortexed to mix. In each well, 144 μl of the above reaction mix and 16 ul of cells were combined. The microtiter plate was sealed and vortexed for 10 seconds, then equilibrated at room temperature for 5 minutes before addition of 50 μl 4 mg/ml 2-nitrophenyl β-D-galactopyranoside (ONPG). When a significant yellow color developed, 90 μl stop solution (1 M Na_2_CO_3_) was added and time was recorded. Reactions were read at A_420 _and A_550_, soon after stopping the reaction; cell density was read at A_600_. Miller Units were calculated as follows: 1000 * ((A_420 _- (1.75*A_550_))/(time (in minutes) * 0.1 * A_600_)). An average of triplicate wells is reported for each sample.

### Analysis of anthranilate and benzoate concentrations

HPLC analyses of culture supernatant was carried out on a Hewlett-Packard Series 1100 HPLC using an isocratic method capable of separating anthranilate, benzoate, catechol, 6-chloroanthranilate and *o*-toluate. The separation column was a ZORBAX Eclipse XDB-C8 (4.6 × 150 mm, 5 μm; Agilent P/N 993967-906) equipped with a Supelguard Discovery C18 guard column (4 × 20 mm; Supelco P/N 505129). The mobile phase contained 25% acetonitrile in 25 mM sodium dihydrogen phosphate (NaH_2_PO_4_), pH 2.5, and the flow rate was 1.0 mL/min. The method used a standard sample injection volume of 2.5 μL and compounds were detected by monitoring absorbance at 254 nm. Under these conditions, compound retention times were as follows: catechol (2.20 min), 6-chloroanthranilate (3.01 min), anthranilate (3.46 min), benzoate (4.98 min), and *o*-toluate (6.53 min). Area responses were linear (R^2 ^0.99) for calibration standards ranging from 0.5 to 5.0 mM.

### RNA Isolation

An overnight culture of MB101 carrying the appropriate plasmid was grown in 1× M9 supplemented with 1% glucose (w/v), 1 mM MgSO_4 _and trace elements. The culture was induced with 5 mM benzoate or anthranilate as appropriate for 8 or 24 hours. Cells were pelleted and total RNA isolated using RNeasy maxi kit (Qiagen). The RNA was resuspended to a final volume of 200 μl and treated with 10 units DNAseI (RNase free) (Ambion) according to manufacturer's protocol. Following DNAseI treatment, the RNA was purified using an RNeasy midi or mini column (Qiagen). RNA concentration was determined using Ribogreen (Molecular Probes).

### Primer extension

Primer Labeling:1 μl 10 μM primer (either lacZPE or lacZPE2), 1 μl 10× T4 kinase buffer, 5 μl ^32^P γ ATP (50 uCi, Amersham Biosciences, Pistcataway, NJ), 1 μl T4 kinase, 2 μl ddH_2_O, was incubated at 37°C for 30–60 minutes. 5 μl of the reaction was reserved to use for sequencing ladder. 20 μl TE was added to the remaining 5 μl and purified using a G25 sephadex column (Amersham-Pharmacia) to remove unincorporated nucleotides, yielding a final concentration of 0.2 μM labelled primer.

Sequencing ladder: The Promega fmol kit, along with 1 μM labelled primer was used to generate a sequencing ladder. Plasmid template used corresponds with that contained in the strain from which RNA was isolated for the extension reaction.

Primer Extension reaction: 10–20 μg of total RNA was mixed with 0.2 pmol primer to yield a final volume of 12 μl, and incubated at 70C for 10 min. To this was added 4 μl 5× Superscript II buffer, 2 μl 1 M DTT, 1 μl 10 mM dNTPs, 1 μl Superscript II or Thermoscript Reverse transcriptase (Invitrogen) and incubated 42°C (Superscript) or 55°C (Thermoscript) for 1 hour. 4 μl of sequencing stop solution was added to the reaction. All reactions were heated at 70°C for 2 minutes immediately before being loaded onto a 6% Long Ranger (Biowhittaker, Rockland, ME)/8 M urea/1.2× TBE gel next to the sequencing ladder. The gel was run in 0.6× TBE buffer, then dried, exposed to phophor screen, and imaged on the Typhoon phophorimager (Molecular Dynamics).

### Inactivation of benzoate transcriptional activator

A DNA fragment containing a portion of the open reading frame (ORF) upstream of the *benA *gene was amplified by PCR using MB214 genomic DNA as template with the benactKO-for and benactKO-rev primers. JM109 was transformed with the resulting product cloned into the pCR2.1 TOPO vector (Invitrogen). Transformants were screened for insert by colony PCR using M13F and M13R primers, and the positive clones were further confirmed by DNA sequencing. The resulting plasmid, pDOW1131, was used to insertionally inactivate the corresponding ORF. *P. fluorescens *strain MB101 was transformed with pDOW1131, selecting on LB agar supplemented with tetracycline. Primers benactKO-for and either M13F or M13R were used to confirm insertion of the plasmid into the desired ORF by colony PCR. The resulting strain was named DC284.

### Construction of *P. fluorescens *Δ*benAB*Δ*proC*Δ*pyrF*

Following the method previously described for construction of gene deletions in *P. fluorescens *[24], the plasmid pDOW1139 was constructed to facilitate deletion of the *benAB *genes as follows. The 3' portion of the *benR *gene and the 5' portion of the *benC *gene were amplified using MB214 genomic DNA as template. The *benR *region was amplified using primers H3_5'benAKOclean and benKOmega. The *benC *region was amplified using primers H3_3'benBKOclean and invbenKOmega. The *benR *and *benC *fragments were fused using primers H3_5'benAKOclean and H3_3'benBKOclean with both fragments as template. The expected 1.1 kb fragment was gel-purified using Qiaex II (Qiagen) and cloned into Srf I digested pDOW1261-2[24]. *P. fluorescens *MB101 derivative DC164 [24] was transformed with the resulting plasmid, pDOW1139. Transformants were selected by plating on LB-proline-uracil with tetracycline for selection. Since the plasmid could not replicate in *P. fluorescens*, tetracycline resistant colonies isolated following transformation resulted from the plasmid integration into the chromosome. The site of plasmid integration was analyzed by PCR. To obtain strains that have lost the integrated plasmid by recombination between the homologous regions, single colonies of the transformants were inoculated into liquid LB supplemented with 250 μg/mL each proline and uracil (LB-proline-uracil), grown overnight, and then plated onto LB-proline-uracil and 500 μg/mL 5'-fluoroorotic acid (FOA) to counter select for the loss of the plasmid [25]. Isolates having the phenotype expected (i.e., tetracycline sensitive, uracil auxotrophic, and FOA resistant) were selected. DNA from the resulting strain (DC253) was analyzed by PCR to confirm removal of the *benAB *region using primers 5'benA_seq, seq_3'benB, M13R21, 1261-8378F and 1261-103R.

## Authors' contributions

DMR participated in design and coordination of experiments, performed sequence alignments, identified promoter regions and participated in promoter analyses, transcriptional start site analyses, 20 L fermentation analyses and in drafting the manuscript. YS and TCT participated in promoter analyses, and in drafting portions of the manuscript. TCT also participated in anthranilate promoter analysis. YS also constructed and analyzed *benR *deletion mutant. KLH participated in 20 L fermentation analyses and in drafting portions of the manuscript. SMR examined carbon source utilization, developed HPLC methods and drafted a portion of the manuscript. VDL constructed and analyzed the *benAB *deletion mutant and contributed to a portion of the manuscript draft. CHS participated in design of experiments. All authors have read and approved the manuscript.
